# Preclinical assessment of transiently TCR redirected T cells for solid tumour immunotherapy

**DOI:** 10.1007/s00262-019-02356-2

**Published:** 2019-06-18

**Authors:** Nadia Mensali, Marit Renée Myhre, Pierre Dillard, Sylvie Pollmann, Gustav Gaudernack, Gunnar Kvalheim, Sébastien Wälchli, Else Marit Inderberg

**Affiliations:** 1 Department of Cellular Therapy, Department of Oncology, Oslo University Hospital, The Norwegian Radium Hospital, 0379 Oslo, Norway; 2 Department of Cancer Immunology, Institute for Cancer Research, Oslo University Hospital, The Norwegian Radium Hospital, 0379 Oslo, Norway; 3 Faculty of Medicine, University of Oslo, 0316 Oslo, Norway

**Keywords:** T cell receptor, mRNA, In vivo model, Solid tumour, Immunotherapy

## Abstract

Off-target toxicity due to the expression of target antigens in normal tissue or TCR cross-reactivity represents a major risk when using T cell receptor (TCR)-engineered T cells for treatment of solid tumours. Due to the inherent cross-reactivity of TCRs it is difficult to accurately predict their target recognition pre-clinically. It has become evident that direct testing in a human being represents the best evaluation of the risks. There is, therefore, a clear unmet need for assessing the safety of a therapeutic TCR in a more controllable manner than by the injection of permanently modified cellular products. Using transiently modified T cells combined with dose escalation has already been shown feasible for chimeric antigen receptor (CAR)-engineered T cells, but nothing is yet reported for TCR. We performed a preclinical evaluation of a therapeutic TCR transiently expressed in T cells by mRNA electroporation. We analyzed if the construct was active in vitro, how long it was detectable for and if this expression format was adapted to in vivo efficacy assessment. Our data demonstrate the potential of mRNA engineered T cells, although less powerful than permanent redirection, to induce a significant response. Thus, these findings support the development of mRNA based TCR-therapy strategies as a feasible and efficacious method for evaluating TCR safety and efficacy in first-in-man testing.

**Electronic supplementary material:**

The online version of this article (10.1007/s00262-019-02356-2) contains supplementary material, which is available to authorized users.

## Introduction

T cell therapy has demonstrated impressive clinical results and is attracting considerable interest in the treatment of both haematological and solid cancers. T cells can be redirected against tumour either by the introduction of a chimeric antigen receptor (CAR) targeting surface antigens or by introducing an α/β T cell receptor (TCR) [1]. CAR T cell treatment against haematological malignancies is so far the most widely used as demonstrated by the recent FDA approval of CD19 CAR T cells for the treatment of B-cell acute lymphoblastic leukaemia (ALL). Both CAR and TCR expression and functionality upon mRNA transfection has been previously shown in vitro [2, 3], in murine hematological xenograft models for CARs [4–8] and recently in models of solid tumour [9] and clinically [10, 11]. TCR mRNA transfection has been demonstrated to be an effective means of screening TCRs for their in vitro efficacy against cancer and infectious disease [12–14], but has to our knowledge never been tested in vivo.

Recent reports of toxicity in clinical trials due to off-target effects of T cells genetically modified to stably express TCRs have increased the interest in using transiently redirected T cells for first-in-man clinical studies [15, 16]. Indeed, there are currently no precise methods to predict the safety of a TCR [17]. Once injected into a patient, the proliferation of activated permanently redirected T cells is very hard to stop, independently of whether they recognize the cancer target or healthy tissues. This is a major issue and transient expression of novel receptors appears as the safest solution to detect off-target events with yet limited risk for the patients.

We have recently shown that a TCR named Radium-1 targeting a neoantigen frequently expressed in microsatellite instable (MSI) colon cancer can be expressed transiently by mRNA electroporation and permanently using retroviral transduction [18]. This TCR recognizes a frameshift mutation in the transforming growth factor β receptor II (TGFβRII) present in the majority of MSI+ colon cancers [19]. In a murine xenograft model of colon cancer, we previously demonstrated the efficacy of stably TCR-engineered T cells in reducing tumour growth and improving survival [18].

We currently have the approval for first-in-man testing of transient TCR therapy (NCT03431311) and we present here the preclinical evaluation of Radium-1 mRNA electroporated T cells. In this study, we confirm our previous observation of the in vitro potency of the Radium-1 TCR to redirect donor T cells and we further assess the in vivo efficacy of these cells in a mouse xenograft model of MSI+ colon cancer. Together our data show the first preclinical assessment of transiently TCR redirected T cells.

## Materials and methods

### Cell lines, media and reagents

T cells were isolated from the blood of healthy donors. Epstein–Barr-Virus-transformed lymphoblastoid cell lines (EBV-LCLs) used as target cells were generated by immortalization of B cells from HLA-A2+ donors using EBV supernatant from the marmoset cell line B95.8. The colon cancer cell line HCT 116 was obtained from ATCC (Rockville, MD, USA). The HCT 116 cell line had also been modified to express EGFP-ffLuc as previously described [18]. Cells were cultured in RPMI-1640 (Gibco, Thermo Fisher Scientific, USA) supplemented with gentamicin and 10% heat-inactivated fetal calf serum (FCS) (Gibco, Thermo Fisher Scientific, USA).

All T cells were grown in CellGro DC medium (CellGenix GmbH, Germany) supplemented with 5% heat-inactivated human pooled serum (TCS Biosciences Ltd, UK), 10 mM *N*-acetylcysteine (Mucomyst 200 mg/ml, AstraZeneca AS, UK), 0.01M HEPES (Life Technologies, Norway) gentamycin 0.05 mg/ml (Garamycin, Schering-Plough Europe, Belgium), and 100 U/ml interleukin-2 (IL-2) (Proleukin, Novartis Healthcare, USA) denoted complete medium hereafter, unless otherwise stated.

### In vitro mRNA transcription of TCR targeting TGFβRII

A TGFβRII frameshift mutation-specific, human leukocyte antigen (HLA)-A2 restricted TCR was identified in a T cell clone from a vaccinated MSI+ colon cancer patient and named Radium-1 [18]. The in vitro mRNA synthesis was performed essentially as previously described [20]. Anti-Reverse Cap Analog (Trilink Biotechnologies Inc., USA) was used for RNA capping. The mRNA quality was assessed by agarose gel electrophoresis and Nanodrop (Thermo Fisher Scientific, USA).

### In vitro expansion of human T cells

T cells from healthy donors were expanded using a protocol adapted for GMP production of T cells employing Dynabeads CD3/CD28 (CTS™ Dynabeads™ CD3/CD28, kindly provided by Gibco, Life Technologies AS, Norway) essentially as previously described [20]. In brief, PBMCs were isolated from buffy coats by density gradient centrifugation and cultured with Dynabeads at a 3:1 ratio in complete CellGro DC medium supplemented with 100 U/ml recombinant human IL-2 (Proleukin, Novartis Healthcare, USA). Fresh complete medium was added regularly and after 10 days expansion CD4^+^ and CD8^+^ T cells were either electroporated together or CD8^+^ T cells isolated as described below.

### CD8 T cell isolation

The CD8^+^ population of in vitro expanded T cells was isolated using the Dynabeads^®^ CD8 Positive Isolation Kit (Invitrogen by Life technologies AS, Norway) following the manufacturer’s protocol. The remainder of T cells were used as the CD4^+^ T cell population. The purity was assessed by flow cytometry and found to be around 98%. The CD8^+^ and CD4^+^ T cell populations were then electroporated separately and used for further experiments.

### Electroporation of expanded T cells

The expanded T cells were washed twice and resuspended in CellGro DC medium (CellGenix GmbH, Germany) at 7 × 10^7^ cells/ml. 100 μg/ml mRNA encoding the TCR or sterile water for mock electroporations was mixed with the cell suspension, and electroporated in a 4-mm gap cuvette at 500 V and 2 ms using a BTX 830 Square Wave Electroporator (BTX Technologies Inc., USA). Immediately after transfection, the T cells were transferred to complete culture medium at 37 °C in 5% CO_2_ overnight to allow TCR expression. The T cells were then used fresh or frozen in aliquots.

### In vitro TCR expression, cytokine production and degranulation measured in flow cytometry

For the functional testing of T cells, the electroporated T cells were either used fresh or thawed. For staining of TCR expression, the T cells were washed in staining buffer (SB) consisting of phosphate buffered saline (PBS) containing 0.1% human serum albumin (HSA) and 0.1% sodium azide before antibody staining for 20 min at room temperature (RT). The cells were then washed in SB and fixed in SB containing 1% paraformaldehyde.

For intracellular staining of cytokine production and degranulation (CD107a) studies, the target cells were loaded or not with peptide 621 (p621), KSLVRLSSCVPVALMSAMT (amino acid sequence 127–145) from a TGFβRII frameshift protein resulting from a 1 bp-deletion (−1A) in an adenosine stretch (A10) from base number 709–718 of *TGFBRII*. (GenBank sequence for wild type human *TGFBRII*: NM 003242) was provided by Norsk Hydro ASA, Norway. Peptide 621 contains the nonameric HLA-A2 epitope (RLSSCVPVA) that binds the Radium-1 TCR previously shown to activate Radium-1 redirected T cells. The 19-mer peptide was preferred for stimulation due to its requirement for processing. For cytokine production and CD107a measurements, T cells were stimulated for 6 h with antigen presenting cells (APC), loaded or not with 1 μM p621, at an effector to target (E:T) ratio of 1:2 and in the presence of 40 μl/ml CD107a-PE-Cy5 antibody (BD Biosciences), and BD GolgiPlug and BD GolgiStop at recommended concentrations. Cells were stained extracellularly and intracellularly for 20–30 min at RT using the PerFix-nc kit according to the manufacturer’s instructions (Beckman Coulter Inc., USA). The following antibodies were used: Vβ3-FITC (Beckman Coulter-Immunotech SAS, France), CD4-BV421 (BioLegend, USA), CD8-PE-Cy7, CD107a-PE-Cy5 (BD Biosciences, USA), interferon gamma (IFN-γ)-FITC, tumour necrosis factor alpha (TNF-α)-PE (BD Biosciences, USA). All antibodies were purchased from eBioscience, USA, except where noted and used at recommended concentrations. The cells were acquired on a BD LSR II flow cytometer and the data analyzed using FlowJo software (Treestar Inc., Ashland, OR, USA).

### Bioluminescence-based cytotoxicity assay

Luciferase-expressing tumour cells, loaded or not with p621 (1 μM), were counted and resuspended at a concentration of 3 × 10^5^ cells/ml. The cells were given Xenolight d-Luciferin potassium salt (75 µg/ml; Perkin Elmer, Norway) and were placed in 96-well white flat bottomed plates at 100 µL cell suspension/well (3 × 10^4^ cells/well) in triplicates. Electroporated effector T cells from healthy donors were used either fresh or thawed and added at indicated E:T ratios. For the determination of spontaneous and maximal killing, wells with the target cells only or with the target cells in 1% Triton™ X-100 (Sigma-Aldrich, Norway) were seeded. Cells were left at 37 °C and the bioluminescence (BLI) was measured with a luminometer (VICTOR Multilabel Plate Reader) as relative light units (RLU) at indicated time points. Target cells incubated without any effector cells were used to determine baseline spontaneous death RLU for each time point. Triplicate wells were averaged and percentage lysis was calculated using following equation: % specific lysis = 100 × (spontaneous cell death RLU − sample RLU)/(spontaneous death RLU − maximal killing RLU). Sigmoid curves (no Hill equation) were fitted for every set of points (using Igor Pro 6.36) as guide for the eye with standard deviation as weighting factor, base hold to 0 and max lysis kept below 100.

### Mouse xenograft studies

NOD.Cg-Prkdc^scid^ Il2rg^tm1Wjl^/SzJ(NSG) mice were bred in-house under an approved institutional animal care protocol and maintained under pathogen-free conditions. 6–8-week-old female mice were injected in intra-peritoneally (i.p.) with 1–1.5 × 10^6^ HCT 116 tumour cells. The HCT 116 cells were engineered with a retroviral vector (provided by Dr. Rainer Löw, EUFETS AG, Germany) to express firefly luciferase and EGFP [18]. Tumour growth was monitored by bioluminescent imaging using the Xenogen Spectrum system and LivingImage v3.2 software. Anaesthetized mice were injected i.p. with 150 mg/kg body weight of d-luciferin (Caliper Life Sciences, Hopkinton, MA). The animals were imaged 10 min after luciferin injection. The mice were treated either i.p. or intravenously (i.v.) with indicated cell numbers of fresh or thawed TCR mRNA electroporated or mock electroporated T cells at indicated time points.

### Statistical analysis

Continuous data were described with mean and standard deviation. Unless stated otherwise all statistics were made with multi-variated bidirectional Student *t* test. Multi-variated bidirectional Mann–Whitney test was used for analysis of tumour load. Mantel–Haenszel test was used as log-rank estimator for survival curves.*p < 0.05, **p < 0.01, ***p < 0.001. All statistical analyses were performed using R.

## Results and discussion

### In vitro evaluation of T cells transiently expressing Radium-1

**Fig. 1:**
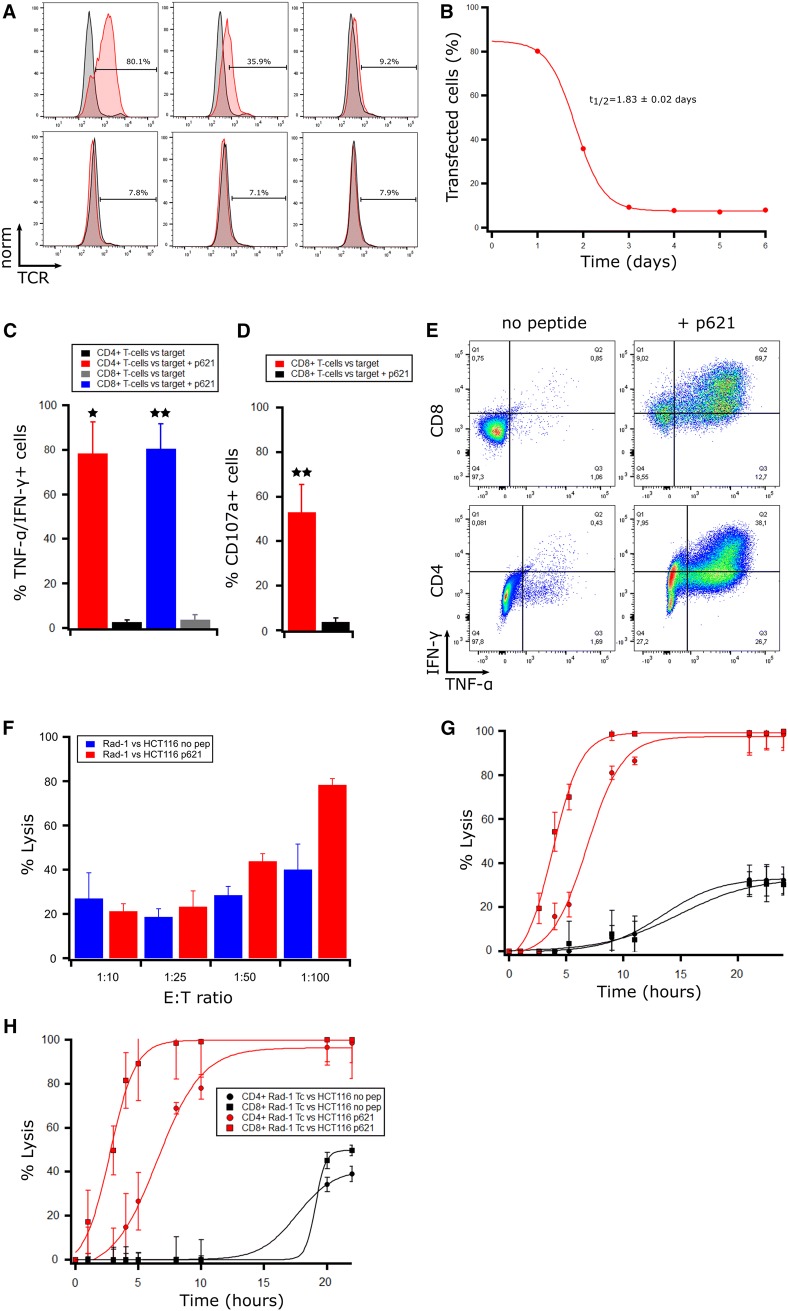
In vitro efficacy of transiently TCR-transfected T cells against targets presenting frameshift mutated TGFβRII in vitro. (**a**) T cells expanded for 10 days with CD3/CD28 Dynabeads were transfected with Radium-1 TCR mRNA. After overnight resting, the TCR expression was detected with a Vβ3-FITC antibody in flow cytometry at several time points post-transfection and compared to mock electroporated T cells. From top left to right; day 1, day 2, and day 3 and from bottom left to right; day 4, day 5, and day 6. (**b**) The graph presents the percentage of TCR transfected T cells with time. **(c**–**e**) The transfected T cells were co-incubated with HLA-A2+ EBV-LCLs loaded (+) or non-loaded (−) with 10 µM frameshift peptide p621. Intracellular cytokine staining (**c**, **e**) and CD107a (**d**) analysis were performed after 6 h incubation (*n* = 3). In vitro expanded T cells from healthy donors were tested for cytotoxicity against luciferase-expressing HCT 116 cells in bioluminescence (BLI) assays at indicated effector:target (E:T) ratios (**f–h**). Radium-1 TCR expressing T cells were tested against luciferase-expressing HCT 116 cells, loaded (red) or not (blue) with p621 (1 µM) at indicated E:T ratios. Mock electroporated T cells from the same donor were used as a negative control and subtracted from the points shown. The graph shows the percentage lysis at 12 h of co-culture (**f**). Radium-1 TCR expressing CD4^+^ and CD8^+^ T cells were tested separately to compare the kinetics of cytotoxicity of the two T cell subsets in two different donors against peptide-loaded HCT 116 cells (**g**, **h**). Radium-1 CD8^+^ T cells (red, squares) and Radium-1 CD4^+^ T cells (red, circles) are shown with their respective mock T cell controls in black

We first tested the expression of the TCR in mRNA electroporated T cells using a Vβ3-FITC antibody as multimers binding the Radium-1 TCR were not available (Fig. 1a). We reproducibly observed that around 75–85% of the total T cells expressed the TCR 18 h after transfection (day 1) and we could further observe expression up to 3–4 days post-electroporation and that the half-life of the TCR was around 2 days (Fig. 1b). The population of T cells endogenously expressing the Vβ3 chain was around 4–7%, depending on the donor. Thus, our protocol is efficient to produce a homogenous population of redirected T cells without any further need to purify them. We previously showed that Radium-1 TCR was partially co-receptor independent when expressed using a retroviral system. We, therefore, studied the functional activity of both CD8^+^ and CD4^+^ T cells after electroporation and found that both T cell subsets produced cytokines (TNF-α and IFN-γ) upon co-incubation with target cells loaded with a 19-mer peptide (p621) containing the 9-mer epitope (Fig. 1c, e). This was important as it suggests that the TCR produced after mRNA electroporation is expressed at sufficient levels to bypass the co-receptor dependency. In addition, CD8^+^ T cells upregulated the degranulation marker CD107a upon target cell recognition (Fig. 1d). We then assessed the cytotoxicity of our total electroporated T cell population against the MSI+ HLA-A2+ HCT 116 colon cancer cell line carrying the TGFβRII frameshift mutation with and without loaded p621 in a BLI based cytotoxicity assay. We found that Radium-1 TCR transfected T cells could kill HCT 116 both in the presence and the absence of exogenously loaded peptide over a range of E:T ratios compared to mock T cells (Fig. 1f). The killing efficiency was higher and killing occurred faster at higher E:T ratios as expected (Supplementary Fig. 1). Several studies have found that CD4^+^ T cells can also be cytotoxic [21–23]. As we planned to use the whole T cell population for T cell redirected therapy, we wanted to test if Radium-1 TCR transfected CD4^+^ T cells were cytotoxic. CD8^+^ and CD4^+^ T cells from two healthy donors were isolated after in vitro T cell expansion and the two T cell subsets were mRNA electroporated separately before testing in cytotoxicity assays against peptide-loaded cancer cells HCT 116 (Fig. 1g, h). Our results confirmed that while overall target cell lysis was similar for CD4^+^ T cells and CD8^+^ T cells, the CD4^+^ sub-population exhibited slower killing kinetics. Whereas CD8^+^ T cells killed 50% of the target cells in approximately 2.5–3 h, CD4^+^ T cells required 7–8 h for the same effect. After approximately 12 h, however, the CD4^+^ T cells had caught up with their CD8^+^ counterparts in both donors.

This was important for our in vivo models where a mix of CD4^+^ and CD8^+^ redirected T cells was infused mainly to test the direct tumour killing efficiency. Taken together, our data demonstrate a level of Radium-1 TCR expression and functionality comparable to that previously observed in retroviral settings, albeit transient.

### In vivo efficacy of transiently transfected T cells comparing intraperitoneal delivery with intravenous administration

We previously demonstrated in our colon cancer xenograft mouse model that stably transduced T cells expressing the Radium-1 TCR significantly reduced the growth of colon cancer [18]. In the current study, we investigated the in vivo efficacy of transiently modified Radium-1 TCR T cells in the same colon cancer model using HCT 116 cell line.

**Fig. 2:**
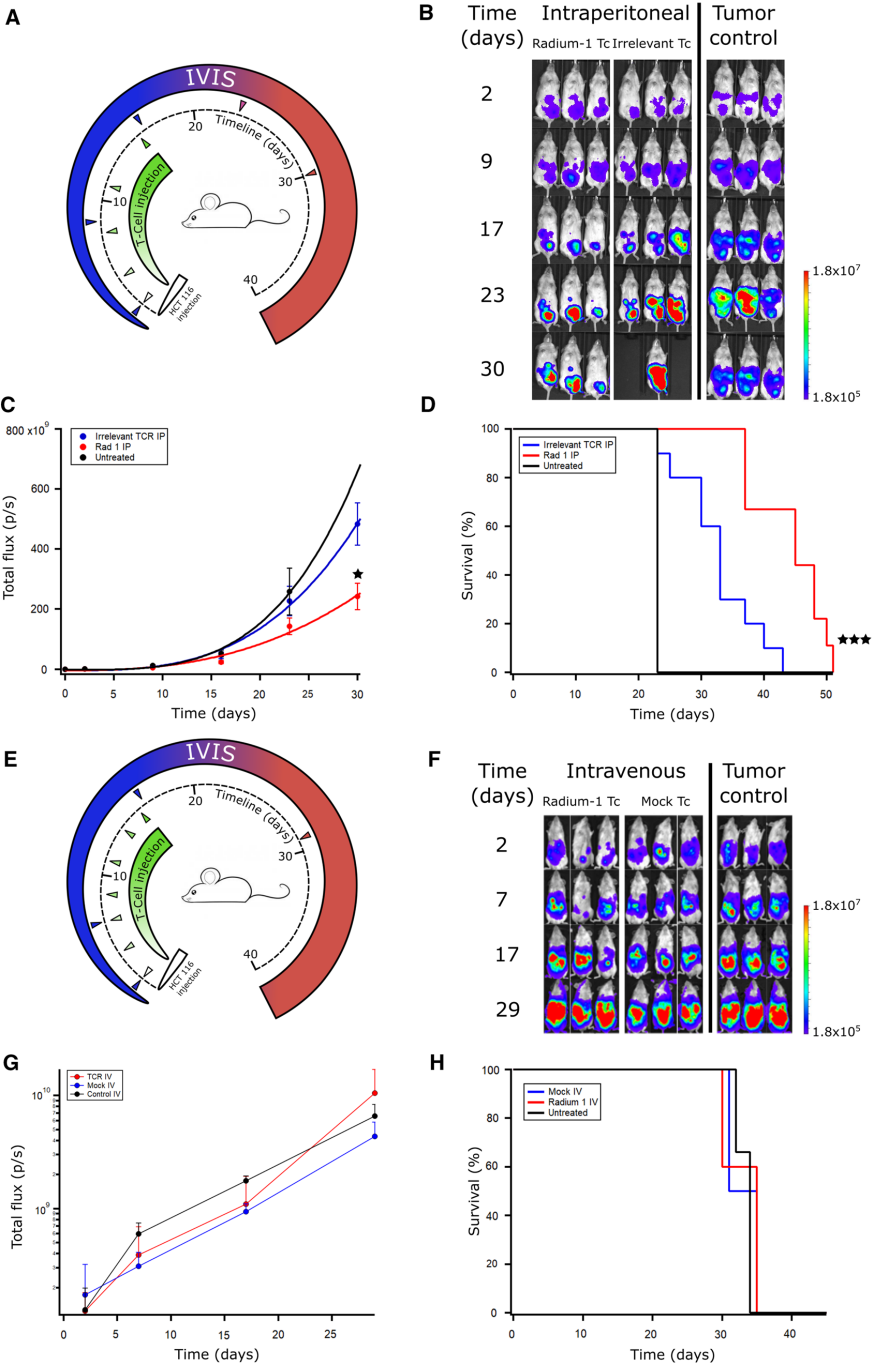
In vivo efficacy of transiently transfected T cells depends on route of administration. (**a**) NSG mice were injected i.p. with 10^6^ HCT 116 ff-Luc 2 days before injection of T cells. T cell treated groups were treated on days 2, 4, 8, 11, and 16 with Radium-1 TCR or irrelevant TCR expressing T cells i.p. (*n* = 10). The Radium-1 TCR group was treated with 10^7^ Radium-1 TCR electroporated T cells at each injection, the irrelevant TCR group (*n* = 10) was treated with 10^7^ DMF5 TCR electroporated T cells at each injection i.p. Tumour control received no treatment (*n* = 4). Three mice from each group are shown. (**b**) Tumour load was evaluated by bioluminescence imaging on days 2, 9, 17, 23, and 30. Black areas indicate loss of mice. (**c**) Average tumour load (total flux photons/s) in the different groups are shown (*p* = 0.031 and 0.0089). (**d**) Survival time of the different treatment groups after tumour injection (*p* = 0.0072). The results shown are representative of three independent experiments. (**e**) Radium-1 Tc treated group (*n* = 5) and mock electroporated Tc treated group (*n* = 4) were treated with 10^7^ T cells i.v. at indicated time points. The tumour control group (*n* = 3) was not given any treatment. (**f**) Tumour load was evaluated by bioluminescence imaging on days 2, 7, 17, and 29. (**g**) Average tumour load (total flux photons/s) in animals given T cells i.v. and control group, (**h**) Kaplan–Meier animals given T cells i.v. and control group

NSG mice were injected intraperitoneally (i.p.) with HCT 116 cells on day 0 (d0) and then treated with multiple infusions of T cells given i.p. on days 2, 4, 8, 11, and 16 (Fig. 2a). The tumour control group received no treatment. Tumour growth was continuously followed using in vivo live imaging (IVIS) (Fig. 2b; Supplementary Fig. 2) and showed a significantly lower tumour burden on day 30 in the mice treated with Radium-1 expressing T cells compared to those carrying an irrelevant TCR (the MART-1 specific DMF5 [24]) (Fig. 2c). The mice treated with the tumour-specific TCR Radium-1 also displayed significantly enhanced survival compared to the two control groups (Fig. 2d).

We also tested the effect of intravenous (i.v.) T cell treatment in this model and infused either Radium-1 electroporated or mock electroporated T cells i.v. on days 2, 4, 7, 9, 11,14, and 16 (Fig. 2e). The tumour control group received no treatment. The tumour growth was followed by IVIS on days 2, 7, 17, and 29 (Fig. 2e, f). Neither mock electroporated nor T cells electroporated with Radium-1 TCR mRNA had any effect on the tumour development or the survival when administered i.v. The mice treated with T cells displayed identical tumour progression as the tumour control group (Fig. 2g, h). This was likely due to the slow trafficking of the transiently modified T cells to the tumour site and consequently with reduced TCR expression. Whether this result is due to working in a xenograft system or if this could be the case in a clinical setting is unknown. Nevertheless, in a recent phase I clinical study, mRNA electroporation was used to transiently redirect T cells with a mesothelin-specific CAR treating six patients with pancreatic cancer [25]. The CARTmeso cells could not be detected in tumour biopsies a few days after the last infusion, but induced humoral epitope spreading against multiple proteins, including immunoregulatory molecules such as PD-1, PD-L1, and BCMA.

In our xenograft model, transiently TCR-redirected T cells had a significant effect on tumour development when delivered to the tumour site i.p. (Fig. 2b–d). At the end of the treatment, the animals treated with T cells expressing Radium-1 TCR displayed a persistent reduction of tumour load compared to the animals that were given irrelevant TCR T cells (*p* = 0.042). This was confirmed in three independent experiments. The survival of the treated animals corresponded to the tumour load and was enhanced for the group of mice treated i.p. with TCR-redirected cells (*p* = 0.0003).

In summary, our results show for the first time that T cells transiently redirected with TCR display antitumour efficacy in a xenograft tumour model. These data support the use of mRNA electroporation for first-in-man clinical testing to minimize the risk of potential toxicity. Furthermore, our results indicate the potential antitumour activity of transiently TCR-redirected T cells against solid tumour. In a clinical setting, such treatment could produce a vaccine effect through tumour cell killing causing the release of tumour-associated antigens. Although from a manufacturing point of view the mRNA product is different from the virally transduced one, the antigen receptor is the same and the present data confirm our previous preclinical observations obtained using a retroviral system. This method would, therefore, give valuable information about the potential danger of a TCR product in very early clinical testing. If proven safe in a transient setting with large cell numbers, this would more easily allow for clinical testing of T cells stably redirected with novel antigen receptors from both a safety and a regulatory perspective.

## Electronic supplementary material

Below is the link to the electronic supplementary material.

Supplementary material 1 (PDF 2513 kb)

## Abbreviations

ALL: Acute lymphoblastic leukaemia
APC: Antigen presenting cells
ATCC: American Type Culture Collection
BCMA: B-Cell maturation antigen
BLI: Bioluminescence
CAR: Chimeric antigen receptor
EBV-LCL: Epstein–Barr-virus-transformed lymphoblastoid cell lines
FCS: Fetal calf serum
FDA: Food and Drug Administration
GMP: Good manufacturing practice
HLA: Human leukocyte antigen
HS: Human serum
IFN-γ: Interferon gamma
IL-2: Interleukin-2
MART-1: Melanoma antigen recognized by T cells 1
MSI: Microsatellite instability
PBMC: Peripheral blood mononuclear cells
PBS: Phosphate buffered saline
PD-1: Programmed cell death protein 1
PD-L1: Programmed cell death ligand 1
PFA: Paraformaldehyde
RT: Room temperature
SB: Staining buffer
TCR: T cell receptor
TGFβRII: Transforming growth factor β receptor II
TNF-α: Tumour necrosis factor alpha

## References

[1] Morris EC, Stauss HJ (2016). Optimizing T-cell receptor gene therapy for hematologic malignancies. Blood.

[2] Birkholz K, Hofmann C, Hoyer S, Schulz B, Harrer T, Kampgen E, Schuler G, Dorrie J, Schaft N (2009). A fast and robust method to clone and functionally validate T-cell receptors. J Immunol Method.

[3] Birkholz K, Hombach A, Krug C (2009). Transfer of mRNA encoding recombinant immunoreceptors reprograms CD4+ and CD8+ T cells for use in the adoptive immunotherapy of cancer. Gene Ther.

[4] Zhao Y, Moon E, Carpenito C (2010). Multiple injections of electroporated autologous T cells expressing a chimeric antigen receptor mediate regression of human disseminated tumor. Cancer Res.

[5] Barrett DM, Zhao Y, Liu X, Jiang S, Carpenito C, Kalos M, Carroll RG, June CH, Grupp SA (2011). Treatment of advanced leukemia in mice with mRNA engineered T cells. Hum Gene Ther.

[6] Kenderian SS, Ruella M, Shestova O (2015). CD33-specific chimeric antigen receptor T cells exhibit potent preclinical activity against human acute myeloid leukemia. Leukemia.

[7] Almasbak H, Walseng E, Kristian A (2015). Inclusion of an IgG1-Fc spacer abrogates efficacy of CD19 CAR T cells in a xenograft mouse model. Gene Ther.

[8] Tasian SK, Kenderian SS, Shen F (2017). Optimized depletion of chimeric antigen receptor T cells in murine xenograft models of human acute myeloid leukemia. Blood.

[9] Hung CF, Xu X, Li L (2018). Development of anti-human mesothelin-targeted chimeric antigen receptor messenger RNA-transfected peripheral blood lymphocytes for ovarian cancer therapy. Hum Gene Ther.

[10] Beatty GL, Haas AR, Maus MV (2014). Mesothelin-specific chimeric antigen receptor mRNA-engineered T cells induce anti-tumor activity in solid malignancies. Cancer Immunol Res.

[11] Tchou J, Zhao Y, Levine BL (2017). Safety and efficacy of intratumoral injections of chimeric antigen receptor (CAR) T cells in metastatic breast cancer. Cancer Immunol Res.

[12] Harrer DC, Simon B, Fujii SI (2017). RNA-transfection of gamma/delta T cells with a chimeric antigen receptor or an alpha/beta T-cell receptor: a safer alternative to genetically engineered alpha/beta T cells for the immunotherapy of melanoma. BMC Cancer.

[13] Mummert C, Hofmann C, Huckelhoven AG, Bergmann S, Mueller-Schmucker SM, Harrer EG, Dorrie J, Schaft N, Harrer T (2016). T-cell receptor transfer for boosting HIV-1-specific T-cell immunity in HIV-1-infected patients. AIDS.

[14] Campillo-Davo D, Fujiki F, Van den Bergh JMJ (2018). Efficient and non-genotoxic RNA-based engineering of human T cells using tumor-specific T cell receptors with minimal TCR mispairing. Front Immunol.

[15] Linette GP, Stadtmauer EA, Maus MV (2013). Cardiovascular toxicity and titin cross-reactivity of affinity-enhanced T cells in myeloma and melanoma. Blood.

[16] Morgan RA, Chinnasamy N, Abate-Daga D (2013). Cancer regression and neurological toxicity following anti-MAGE-A3 TCR gene therapy. J Immunother.

[17] Kunert A, Obenaus M, Lamers CHJ, Blankenstein T, Debets R (2017). T-cell receptors for clinical therapy: in vitro assessment of toxicity risk. Clin Cancer Res.

[18] Inderberg EM, Walchli S, Myhre MR, Trachsel S, Almasbak H, Kvalheim G, Gaudernack G (2017). T cell therapy targeting a public neoantigen in microsatellite instable colon cancer reduces in vivo tumor growth. Oncoimmunology.

[19] Schwitalle Y, Kloor M, Eiermann S, Linnebacher M, Kienle P, Knaebel HP, Tariverdian M, Benner A, von Knebel Doeberitz M (2008). Immune response against frameshift-induced neopeptides in HNPCC patients and healthy HNPCC mutation carriers. Gastroenterology.

[20] Almasbak H, Rian E, Hoel HJ, Pule M, Walchli S, Kvalheim G, Gaudernack G, Rasmussen AM (2011). Transiently redirected T cells for adoptive transfer. Cytotherapy.

[21] Patil VS, Madrigal A, Schmiedel BJ (2018). Precursors of human CD4(+) cytotoxic T lymphocytes identified by single-cell transcriptome analysis. Sci Immunol.

[22] Hsu DC, Breglio KF, Pei L (2018). Emergence of polyfunctional cytotoxic CD4+ T cells in mycobacterium avium immune reconstitution inflammatory syndrome in human immunodeficiency virus-infected patients. Clin Infect Dis.

[23] Serroukh Y, Gu-Trantien C, Hooshiar Kashani B (2018). The transcription factors Runx3 and ThPOK cross-regulate acquisition of cytotoxic function by human Th1 lymphocytes. eLife.

[24] Johnson LA, Heemskerk B, Powell DJ, Cohen CJ, Morgan RA, Dudley ME, Robbins PF, Rosenberg SA (2006). Gene transfer of tumor-reactive TCR confers both high avidity and tumor reactivity to nonreactive peripheral blood mononuclear cells and tumor-infiltrating lymphocytes. J Immunol.

[25] Beatty GL, O’Hara MH, Lacey SF (2018). Activity of mesothelin-specific chimeric antigen receptor T cells against pancreatic carcinoma metastases in a phase 1 trial. Gastroenterology.

