# Supplementary material for: Preclinical assessment of transiently TCR redirected T cells for solid tumour immunotherapy
Source: Cancer Immunol Immunother. 2019 Jun 18;68(8):1235–43. doi: 10.1007/s00262-019-02356-2 (PMC6682583; doi:10.1007/s00262-019-02356-2)
Supplement: Supplementary file 1 — Supplementary material 1 (PDF 2513 kb) [file 262_2019_2356_MOESM1_ESM.pdf]

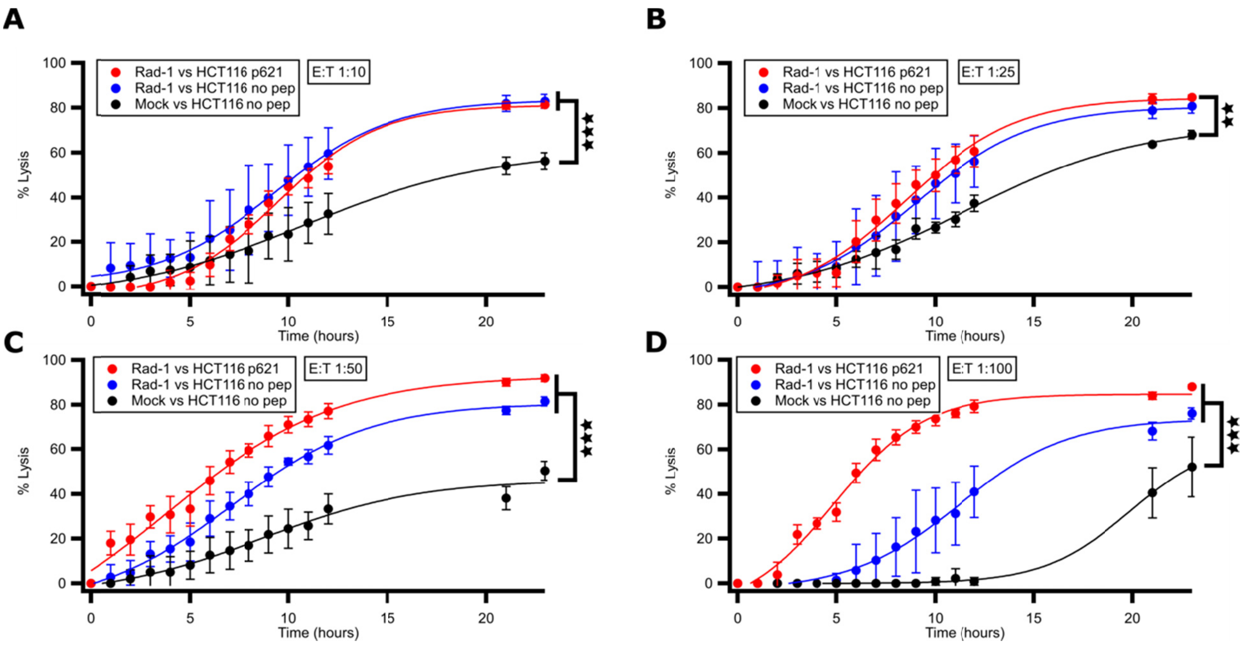

Supplementary fig. 1 TCR mRNA electroporated T cells are capable of lysing tumour cells. *In vitro* expanded and TCR mRNA electroporated T cells from healthy donors were tested for cytotoxicity against luciferase-expressing HCT 116 cells in bioluminescence (BLI) assays at indicated Effector:Target (E:T) ratios **(A-D)** and the cytotoxicity was measured at indicated time points. The HCT 116 target cells were loaded or not with the frameshift peptide p621 (1µM) and the cytotoxicity of Radium-1 TCR expressing T cells was compared against mock electroporated T cells acting as negative controls. Points represent mean ± SD of quadruplicates.

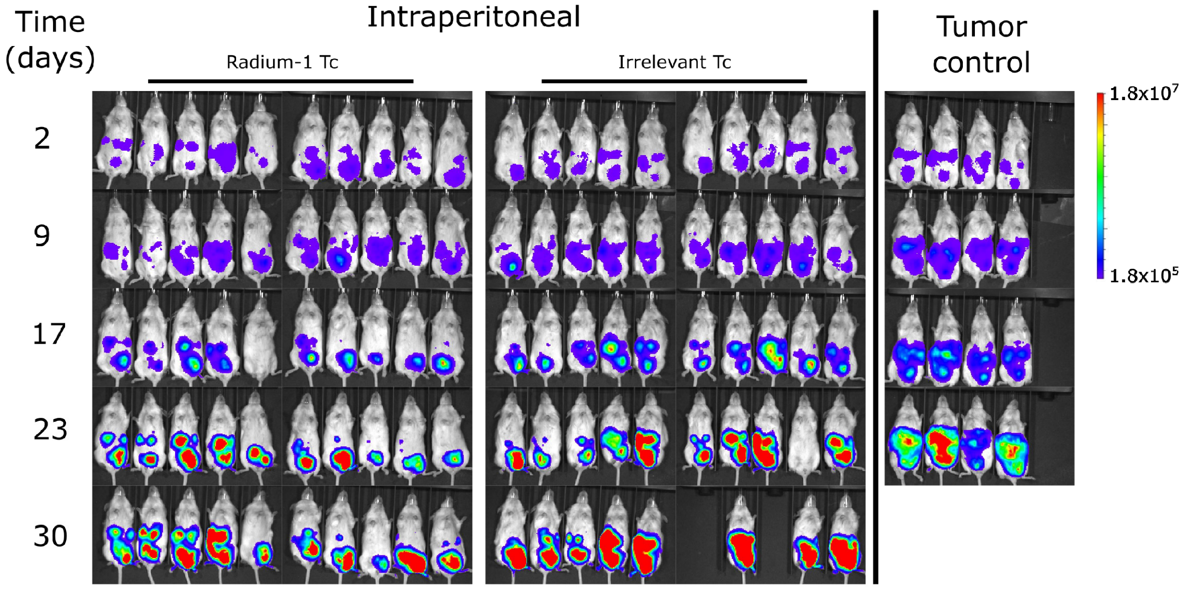

Supplementary fig. 2 Tumour load is reduced in mice treated with Radium-1 TCR mRNA electroporated cells. NSG mice were injected i.p. with 10^6 HCT 116 ff-Luc two days before injection of T cells. T-cell treated groups were treated on days 2, 4, 8, 11, and 16 with 10^7 Radium-1 mRNA electroporated T cells, or irrelevant TCR DMF5 electroporated T cells i.p. The tumour control group received no treatment (n=4). The figure shows all mice included in the experiment in Fig. 2 A-D.
